# Expanding Imaging Capabilities for Microfluidics: Applicability of Darkfield Internal Reflection Illumination (DIRI) to Observations in Microfluidics

**DOI:** 10.1371/journal.pone.0116925

**Published:** 2015-03-06

**Authors:** Yoshihiro Kawano, Chino Otsuka, James Sanzo, Christopher Higgins, Tatsuo Nirei, Tobias Schilling, Takuji Ishikawa

**Affiliations:** 1 The Department of Biomedical Engineering, Graduate School of Biomedical Engineering, Tohoku University, Sendai, Miyagi, Japan; 2 Olympus Corporation, Shinjuku-Ku, Tokyo, Japan; 3 Olympus Scientific Solutions Americas, Waltham, Massachusetts, United States of America; 4 Olympus Engineering, Hachioji, Tokyo, Japan; 5 Olympus Soft Imaging Solutions GmbH, Münster, Germany; 6 Department of Bioengineering and Robotics, Tohoku University, Sendai, Miyagi, Japan; University of Illinois at Chicago, UNITED STATES

## Abstract

Microfluidics is used increasingly for engineering and biomedical applications due to recent advances in microfabrication technologies. Visualization of bubbles, tracer particles, and cells in a microfluidic device is important for designing a device and analyzing results. However, with conventional methods, it is difficult to observe the channel geometry and such particles simultaneously. To overcome this limitation, we developed a Darkfield Internal Reflection Illumination (DIRI) system that improved the drawbacks of a conventional darkfield illuminator. This study was performed to investigate its utility in the field of microfluidics. The results showed that the developed system could clearly visualize both microbubbles and the channel wall by utilizing brightfield and DIRI illumination simultaneously. The methodology is useful not only for static phenomena, such as clogging, but also for dynamic phenomena, such as the detection of bubbles flowing in a channel. The system was also applied to simultaneous fluorescence and DIRI imaging. Fluorescent tracer beads and channel walls were observed clearly, which may be an advantage for future microparticle image velocimetry (μPIV) analysis, especially near a wall. Two types of cell stained with different colors, and the channel wall, can be recognized using the combined confocal and DIRI system. Whole-slide imaging was also conducted successfully using this system. The tiling function significantly expands the observing area of microfluidics. The developed system will be useful for a wide variety of engineering and biomedical applications for the growing field of microfluidics.

## Introduction

Micro total analysis systems (μTASs) have become popular over the last two decades [1]. Microscale tools to control fluid flow, such as micro valves, pumps, and flow sensors have been developed [2]. Microfluidics technologies are now capable of manipulating nanoliters of fluid, molecules, bubbles, particles, and cells [3], and these have been used for micro-fabrication in engineering settings as well as diagnosis in clinical settings [4]. Recently, techniques for producing microbubbles using microfluidic devices have been developed, and the two-phase flow in microchannels has been measured [5–12]. Some groups have succeeded in fabricating monodispersed microbubbles for drug delivery, and in clinical diagnosis they have been used as contrast agents. Droplets and vesicles can also be fabricated by microfluidic devices, which can then be used in synthesizing molecules, such as proteins and DNA [10]. To design such microfluidic devices appropriately requires an accurate dynamic analysis of the motion of bubbles and droplets, with high resolution in time and space. In particular, interactions between bubbles and channel walls are important, because microbubbles may block microchannels, which alters the flow compared to what was expected (i.e., clogging). Moreover, bubble shape is important in the breakup and coalescing of microbubbles in microfluidics. Thus, visualization techniques that enable detection of the surfaces of bubbles and walls are important [13–15].

In microfluidics research, the velocity field is often measured using microparticle image velocimetry (μPIV) [16–19]. Recently, confocal laser scanning microscopes (CLSM), such as disk scanning confocal systems, have been adapted to μPIV systems to improve image contrast and reduce the depth of field of the image [20, 21]. Three-dimensional optical sectioning has become possible and semi-3D velocity measurements have been performed [22–30]. To enable highly accurate velocity measurements, high-contrast images of tracer particles are required. Moreover, the wall configuration must be observed clearly to evaluate the wall shear stress, which can be calculated from the velocity gradient observed at the wall. Thus, it is necessary to simultaneously and clearly observe the wall configuration within channels and fluorescent tracer particles flowing through the channels.

Microfluidics technologies have also been used in cancer diagnosis. For example, an antibody-based microfluidics platform can capture cervical cancer cells using immunocytochemistry techniques [31]. Oral squamous cell carcinoma (OSCC) cells were detected with a microfluidic device using magnetic beads conjugated with antibodies plus real-time polymerase chain reaction (RT-PCR) [32]. Microfluidic devices have been used to identify circulating tumor cells (CTCs) in the peripheral blood of patients with metastatic lung, prostate, pancreatic, breast, colon, and ovarian cancers [33–50]. In the design of such microfluidic devices, it is important to observe the behavior of cells within channels with complex geometry. Moreover, cancer cell adhesion and invasion have been investigated using microfluidics to assess the mechanisms of cancer metastasis [51–57]. As the interactions between cancer cells and vessel walls are important, visualization techniques to detect cancer cells and their relationships to channel walls are critical to understanding cell behavior [13–15]. Such techniques are investigated in this study.

The most common illumination systems used for cell and microfluidics observation in previous studies were brightfield, darkfield, fluorescence, and confocal imaging. Regular darkfield illumination is typically used for particle observations [59]. An example is its use in a blood cell counting application [60]. Fluorescence and confocal techniques are useful for observing specific cell markers. However, simultaneous detection of the channel wall and the cell marker is difficult. Lima et al. combined transmitted halogen illumination and confocal imaging to increase the visibility of fluorescently labeled cells and walls [28]. The combined imaging methods were effective, although the fluorescent signal was partly masked by the halogen illumination. In the present study, we overcame this problem by using a Darkfield Internal Reflection Illumination (DIRI) system combined with a spinning disk confocal microscope. Our group recently developed a modification of the darkfield microscopy system [58]. In the previous study, the applicability of using a DIRI for a fixed brain slice sample was investigated [58]. In the present study, we investigated the applicability of fluorescence and DIRI for microfluidic systems containing microbubbles, fluorescent particles, and/or cells. We show that the combined illumination techniques can simultaneously detect the channel wall and the microbubbles, fluorescent particles, and/or cells with a good apparent signal to noise ratio.

Microfluidic devices with multiple reactor areas and/or multiple measurement sites are often larger than the field of view of a typical 20× or 40× microscope objective lens. Thus, whole-slide imaging (WSI) is useful to observe the entirety of these areas. WSI technology is well suited for observing areas larger than one field of view under a microscope, and is used for tile imaging, remote viewing, archiving, and analyzing images for neuroscience and pathology fields. In this study, we adapted WSI technology to microfluidics observation. The proposed methodology should be useful in future for controlling microfluidics by monitoring multiple locations.

The paper is organized as follows. In the Materials and Methods section, we first describe our DIRI system and compare it with conventional darkfield illumination. We then explain the details of experiments using brightfield, DIRI, and fluorescent illumination. In the Results and Discussion section, we first investigate the combination of brightfield and DIRI imaging that enables visualization of both microbubbles and channel walls simultaneously. We show that the methodology is useful for dynamic phenomena, such as the detection of bubbles flowing in a channel. Second, we investigate the combination of fluorescence and DIRI imaging. We show that the present system can detect both the channel wall and fluorescent tracer beads. Third, we investigate the combination of multi-channel fluorescence imaging and DIRI imaging using two types of cells, fluorescently stained red or green. We show that the present system can detect both the channel wall and the target cell marker. Last, we discuss the advantage of our DIRI system. In the Conclusion section, we summarize the study and provide final thoughts.

## Materials and Methods

### 1. Darkfield Internal Reflection Illumination (DIRI)

**Fig 1:**
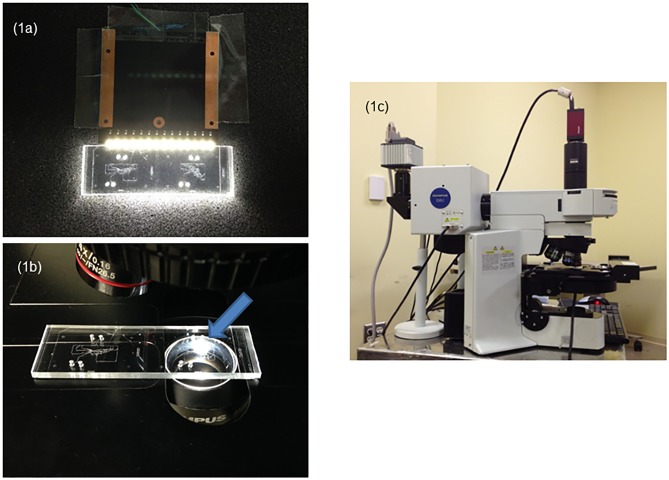
The darkfield illuminator and DIRI illumination areas. (1a) The photograph shows the microfluidic device used with the DIRI illuminator. The illuminated area is almost the entire microfluidic device. (1b) The photograph shows the microfluidic device used with a regular darkfield illuminator. The condenser lens is a DCD condenser, NA 0.8–0.92 (Olympus). The illuminated area is shown by the blue arrow. Note the small area properly covered by the illuminator. (1c) The experimental setup for the system. The left side camera is an Orca R2. The microfluidic device consists of a special microfluidics module placed upon a plastic adapter plate. The size of the microfluidics module in the photograph is 25 × 75 mm.

Fig. 1A shows the newly developed DIRI (Darkfield Internal Reflection Illumination) unit. A multiple LED light assembly is installed on the stage plate and used for side illumination of a microfluidics cell or a glass slide. The DIRI system can be used alone, or may be used with a fluorescence microscope. Acquired fluorescent images are used to show fluorescent markers of target objects. The DIRI system creates a broad scattering of illumination from the edges of the PMDS channel walls. The refractive index mismatch between PMDS and buffer (liquid) produces good image contrast, resulting in high-quality images of the microchannel walls. Superimposition of the two images (fluorescence and DIRI) allows observation of both fluorescent markers and the edges of the channel walls. There are two ways to produce such superimposed images: (a) fluorescence and DIRI images can be acquired simultaneously, or (b) fluorescence and DIRI images can be acquired sequentially as two or more channels (depending on the number of fluorescent channels to be captured separately), and the channels can be superimposed later. Method (a) can be applied to dynamic phenomena as well as static phenomena, whereas method (b) can be applied only to static phenomena.

In the case of conventional darkfield microscopy, light enters the specimen at an oblique angle and is blocked at the periphery of the optical components. As the scattering of light based on the refractive mismatch passes through the optical path and is observed by the darkfield microscope, the background image is dark, and the specimen appears bright. If the image background is bright, the detection sensitivity is considerably reduced, resulting in conditions similar to brightfield microscopy. Darkfield microscopy can often provide enhanced detection sensitivity and contrast for specimens that cannot be effectively imaged using brightfield illumination.

Conventional darkfield units are commercially available for standard microscopes. There are two types of darkfield condenser: dry and oil darkfield condensers. Some phase turret condensers include the required darkfield annulus within the condenser turret. In Fig. 1B, a dry darkfield DCD condenser (NA 0.8–0.92; Olympus, Tokyo, Japan) is shown below the microchannel slide. The illuminated area is limited to a spot (see arrow). Normally, most dry darkfield condensers are capable of illuminating fields of view of 10× to 20× objectives with NA < 0.5. A dry darkfield condenser cannot cover objective lenses with NA > 0.8, such as a 40×, NA 0.95 lens because the illumination cone of light passes directly into the objective lens and eliminates the dark background of the image. Use of the 40×, NA 0.95 objective lens requires optical coverage that can be provided by an oil darkfield condenser. A common practice during imaging sessions is to find the required field of view using a 10× or 20× lens, then to switch to a higher magnification and higher NA objective (such as the 40×, NA 0.95 lens) for image acquisition. However, if the user later needs to switch back to the 20×, NA 0.75 dry lens, the condenser also has to be switched back to the dry darkfield condenser. This necessitates the disruptive and somewhat laborious process of removing oil from the slide, and requiring that the investigator begin again with dry optics. The procedure of switching back and forth between dry and oil systems poses considerable inconvenience to investigators and may introduce unacceptable delay into the experimental protocol, rendering some experiments impossible to perform. In fact, this is the case for many microchannel setups with multiple delicate attachments to pumps, drains, sensors, etc.

As described here, the newly developed DIRI system eliminates the inconvenience and delay inherent in the conventional approach. Thus, experiments can be performed that require frequent or rapid switching of magnification to locate fine details within larger fields of view before acquisition is appropriate. In addition, the area of view that is illuminated for DIRI imaging is larger than that which a conventional darkfield condenser is capable of illuminating (compare Fig. 1A to Fig. 1B). Fig. 1A clearly shows that the DIRI system can illuminate most of the area of a glass slide. The illumination intensity has also been enhanced in this version of the device. The latest design for DIRI is 1.5-fold brighter than the previous design [58]. Moreover, the DIRI can be used with 10× to 100× objective lenses, including both dry and oil objectives. Thus, the DIRI system has important advantages in usability compared to the conventional darkfield condenser.

The DIRI system also has an advantage in terms of the small space required for implementation. The DIRI illuminator is thin and built into the stage insert plate, as shown in Fig. 1A. Therefore, it does not interfere with tubes connected to the microfluidics equipment for liquid handling. Our DIRI is optimized to work with a standard microscope slide measuring 25 × 75 mm. As the size of the microfluidic device used in this study is 25 × 50 mm, we implemented a plastic support plate to adapt the microfluidic device size to 25 × 75 mm. The simplicity of this adaptation is another indication of the robustness and flexibility of our DIRI system as applied to microfluidics.

Our DIRI system is easily installed by simply attaching the LED array to the side of the microfluidics observation location. When we constructed the first prototype, we started from such a simple design. By adding automatic control of the confocal illumination, the widefield fluorescence excitation and emission, DIRI, transmitted (brightfield) illumination, stage movement, etc., a more sophisticated system can be constructed (cf. Fig. 1C).

### 2. Experimental Setup and Procedures

In this study, we conducted three types of experiment: (1) observation of microbubbles by combination of brightfield and DIRI imaging, (2) observation of fluorescent tracer particles by combination of fluorescence and DIRI imaging, and (3) observation of two types of stained cell by combination of multiple fluorescence imaging and DIRI imaging.

In all of these experiments, we used the Virtual Slide System VS120 (Olympus) including VS-ASW WSI software (ver. 2.7) as a base system, with the cellSens software (ver. 1.9; Olympus Soft Imaging Solutions, Münster, Germany) for video and 3D imaging. We attached a BX-DSU spinning disk confocal system (Olympus) to the VS120 system for confocal observations. We also attached the DIRI illuminator as in our previous study [58] for WSI. Before use, this integrated system was calibrated using the VS-ASW software and the recommended calibration slide from the manufacturer. The system described here is a prototype and is not yet commercially available.

**Fig 2:**
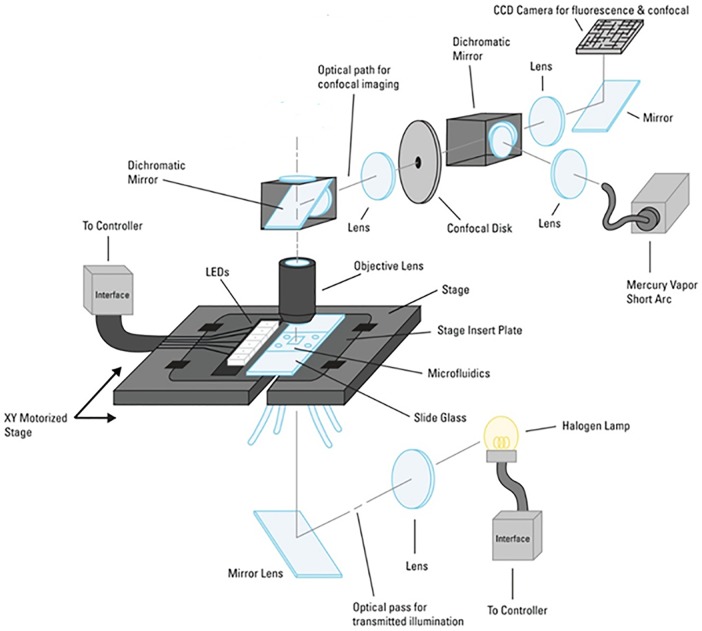
Schematic drawing of a microfluidic observation system. This system is capable of brightfield, DIRI (Darkfield Internal Reflection Illumination), conventional fluorescence, and confocal imaging. The DIRI image was obtained by illumination using the LEDs at the side of the slide. The system can also provide whole-slide images (WSI).

A photograph of the system is shown in Fig. 1C, and the optical layout is shown schematically in Fig. 2. The DIRI system consists of LEDs attached to the microscope stage [58], which enables visualization of the sample by refractive index mismatch. The DIRI system can be used with widefield fluorescence or confocal imaging. A microfluidic device on an appropriate stage adapter is placed on the stage, and the condenser lens for the transmitted illumination is set up about 10 mm below the Köhler illumination position. The condenser lens is located between the stage and mirror unit below the stage (not shown in Fig. 2). The halogen lamp is used for transmitted illumination. The spinning disk confocal system is set between two dichromatic mirrors (cf. Fig. 2). Mercury vapor short-arc lamps are used for confocal and fluorescence illumination. The observed images are recorded with a CCD camera (Orca R2; Hamamatsu Photonics, Hamamatsu, Japan). We typically use the Orca R2 camera for all modes of image capture, including DIRI, fluorescence, confocal, and brightfield imaging.

**Fig 3:**
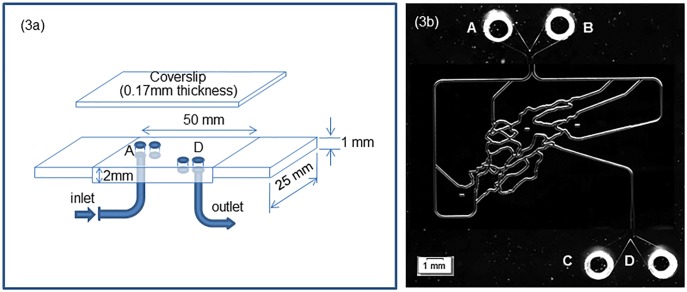
Microfluidic device. (3a) Schematic of the entire plate. The microfluidic module itself was 25 × 50 mm, which was extended to 25 × 75 mm by the addition of a plastic adapter plate. (3b) Channel geometry within the microfluidic device. The image was taken using DIRI (Darkfield Internal Reflection Illumination). Port A is the inflow port, and port D is the outflow port. Ports B and C are closed during the experiment.

Fig. 3 shows the microfluidic device used in this study (SynVivo Bio-chip Microvascular Network; CFD Research Corporation, Huntsville, AL). The original device measures 25 × 50 mm with a thickness of 2 mm (Fig. 3A), and the top surface is covered with a coverslip 0.17 mm thick. The microchannel network is molded into the polydimethylsiloxane (PDMS) block that composes the microchannel device. For this device, the channel width is about 100 μm. Ports A and D in Fig. 3B are the inlet and outlet for fluid flow, respectively. Ports B and C are closed, but are used to remove bubbles in the channel before the experiments. The fluid flow is generated by manipulating a syringe (Norm-Ject, 1 mL; Henke Sass Wolff, Tuttlingen, Germany) with a programmable syringe pump (Model Fusion 200; Chemyx Inc., Stafford, TX). The refractive index of PDMS is approximately 1.41, the buffer has a refractive index similar to water (n = 1. 33), and that of cells is in the range 1.35–1.40. When we used DIRI with buffer-filled channels, scattered light was observed at the interface of the two materials (channel walls) due to their differing refractive indexes.

In the microbubble experiment, we added a diluted surfactant (Triton X-100; Sigma-Aldrich, St. Louis, MO) to phosphate-buffered saline (PBS). The bubbles were generated manually by pushing the syringe plunger back and forth before the experiment. Then, the two-phase fluid was injected into the channel manually using the syringe.

For our fluorescent bead experiments, we added diluted surfactant (Triton X-100; Sigma-Aldrich) to phosphate-buffered saline (PBS) to avoid adhesion of the beads to the channel walls. We used fluorescent beads 1 μm in diameter (F8823 FluoSpheres fluorescent beads; Invitrogen, Carlsbad, CA) diluted with PBS.

In the cell experiments, we used two types of cell emitting two different colors under fluorescence illumination: human umbilical cord blood endothelial cells at the fourth passage (HUVECs, #KE-4109; Kurabo, Osaka, Japan) and fibrosarcoma cells (HT1080; American Type Culture Collection, Manassas, VA). All cells were incubated at 37°C in 5% CO2. The endothelial cells were stained with 10 μg/mL rhodamine 123 (Wako, Osaka, Japan) and emitted green fluorescence when illuminated with blue light. Fibrosarcoma cells were stained using Mito Tracker Red CMXRos (Life Technologies, Gaithersburg, MD) and emitted red fluorescence when illuminated with green light. The numbers of endothelial cells and fibrosarcoma cells were adjusted to 6×105/mL. Before introduction of cells into the microchannel device, the channels were coated with fibronectin to promote adhesion to the channel walls.

In all of these experiments, we used DIRI from only one side of the microfluidic device. Therefore, the DIRI induced asymmetric illumination. More isotropic illumination could be created by the attachment of additional DIRI illuminators located around the periphery, creating concurrent illumination from multiple directions. For example, DIRI illuminators could be attached to both sides of the sample or surrounding the sample.

## Results and Discussion

### 1. Combination of Brightfield and DIRI Imaging

When using a microfluidic device, air bubbles may adhere to the inside of the microchannel, and thus make flow control difficult. Microbubbles are small in size and difficult to find within the entire microfluidic device, so it is important to develop a methodology to detect microbubbles throughout the device. In addition, microbubbles are difficult to detect using standard brightfield illumination because both the bubble signal and the background signal produce images of similar brightness with minimal contrast. In most cases, liquid and air bubbles are both transparent, only differing in refractive index. Darkfield illumination, however, allows the microbubbles to be detected because of the image contrast created by the refractive index differences. Under darkfield illumination, microbubbles appear as bright objects against a dark background. Here, we investigated the utility of our DIRI system for detecting microbubbles.

**Fig 4:**
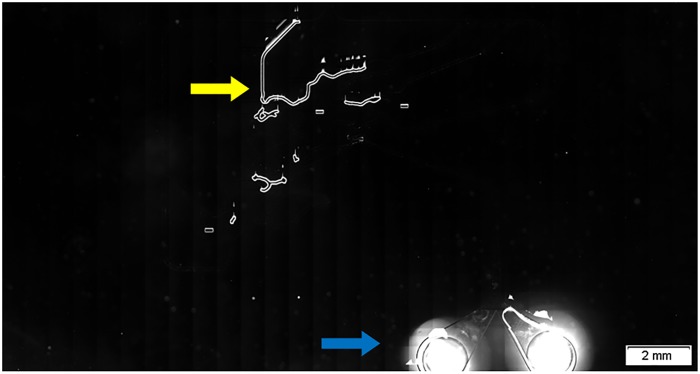
DIRI image of bubbles trapped in the microfluidic channels. Bubbles trapped in the microfluidic channels are indicated by the yellow arrows. The blue arrows indicate outlet ports. Photograph was taken using DIRI (Darkfield Internal Reflection Illumination) with WSI (whole-slide imaging).

Fig. 4 shows a DIRI image of the whole microfluidic device, in which bubbles were observed as bright spots, as indicated in yellow. The four large bright circles indicated by the blue arrows are the inlets and outlets. The VS-ASW software was used to construct a whole-slide image by stitching 1088 individually acquired images. Our system facilitated detection of bubbles in the entire microfluidic device with high contrast.

**Fig 5:**
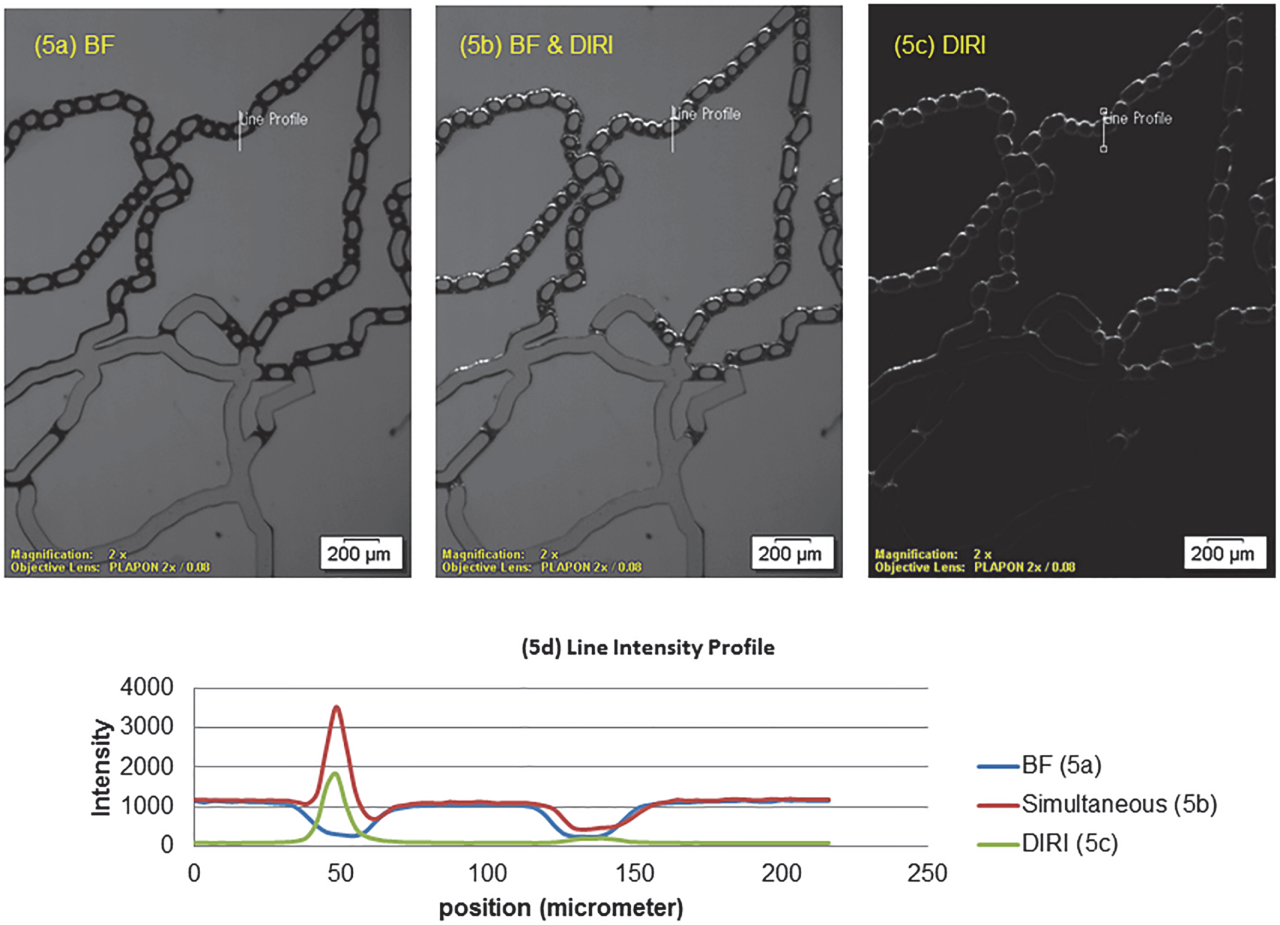
Photographs of bubbles in the microfluidics system taken using brightfield and DIRI. This image was acquired using a 2× objective lens (NA 0.08) and an exposure time of 10 ms. (5a) Photograph obtained with brightfield illumination alone. (5b) Photograph obtained by simultaneous using brightfield illumination and DIRI (Darkfield Internal Reflection Illumination). (5c) Photograph obtained with DIRI alone.

To clarify the advantages of the system, we compared microbubble images using brightfield illumination alone (Fig. 5A), simultaneously used DIRI and brightfield illumination (Fig. 5B), and DIRI alone (Fig. 5C). The images were obtained with a 2× objective lens (NA 0.08), and no flow was induced (i.e., the bubbles were stationary). The brightfield image (Fig. 5A) shows the darker edge of the channel and bubbles on top of a brighter background. The DIRI image (Fig. 5C), however, shows the bright edge of the bubbles on a darker background, although the edge of the channel is unclear. This bright edge of the bubble image is produced by total reflected light from the LEDs of the DIRI. When DIRI and brightfield illumination were used simultaneously (Fig. 5B), the edges of both the channel and bubbles themselves could be observed clearly.

To quantitatively discuss the edge detection sensitivity with different illumination, we measured the intensity profile along a line crossing a bubble and the channel walls, as shown in Fig. 5D. The measured line is indicated as a white line in Fig. 5A—C, which has a length of about 220 μm. In this figure, the intensity varies from 0 to 65535, given that the camera acquired a 16-bit image. As can be seen in the figure, the curve of the line (labeled “Simultaneous”) from the simultaneous illuminated image (5B) shows the greatest variation in brightness at the channel wall and the bubble surface, indicating that the dual illumination technique of brightfield and DIRI is advantageous for simultaneous detection of bubbles and channel edges.

**Fig 6:**
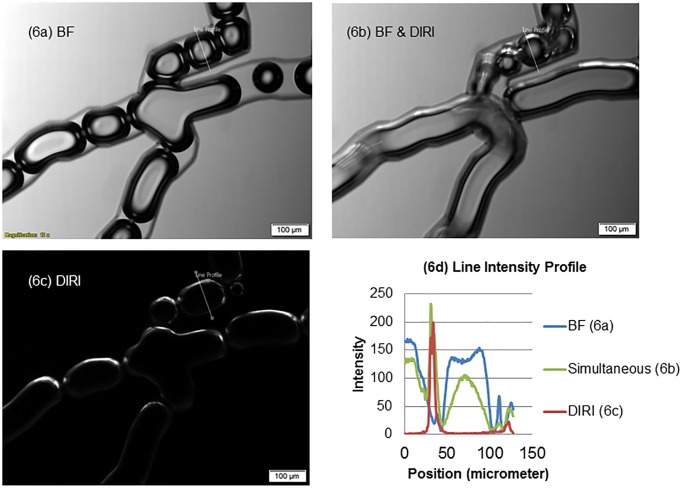
Magnified photographs of bubbles in the microfluidics system using brightfield and DIRI. This image was acquired using a 10× objective lens and an exposure time of 60 ms. (6a) Photograph obtained with brightfield illumination alone. (6b) Photograph obtained by simultaneous using brightfield illumination and DIRI (Darkfield Internal Reflection Illumination). (6c) Photograph obtained with DIRI alone.

Next, we induced fluid flow, to put the bubbles in motion. Although the local velocity fluctuated due to breakup and coalescing of bubbles, the velocity of bubbles in the observed region was on the order of about 10 mm/s. Fig. 6 shows a comparison of microbubble images using three kinds of illumination. Images were acquired with a 10× objective lens (NA 0.4). Fig. 6A—C were acquired with different exposures although the velocity of fluid flow remained constant, so the bubble sizes appear different in each image. The brightfield image (Fig. 6A) clearly shows the edges of the channel walls and the edges of the bubbles. The DIRI image (Fig. 6C) shows total reflected light from the top side at the edges of the bubbles. Note that the edges of the channel walls are difficult to observe. Fig. 6B was taken using brightfield illumination and DIRI simultaneously.

To quantitatively discuss the edge detection sensitivity with different illumination, we again measured the intensity profile along a line crossing the bubbles and the channel walls. The measured line is indicated as a white line in Fig. 6A—C, which has a length of about 130 μm. In this figure, the intensity ranges from 0 to 255, given that the video mode camera setup stored 8 bits of intensity depth. The simultaneous brightfield and DIRI illumination (Fig. 6B) has high contrast at the bubble surfaces as well as the channel wall. This system has the advantage that it can provide a high-contrast image by simultaneously using two kinds of illumination, rather than requiring the superimposition of two images taken sequentially using the two different kinds of illumination. Therefore, this method can be applied to dynamic phenomena, such as gas-liquid two-phase flow.

### 2. Combination of Fluorescence and DIRI Imaging

**Fig 7:**
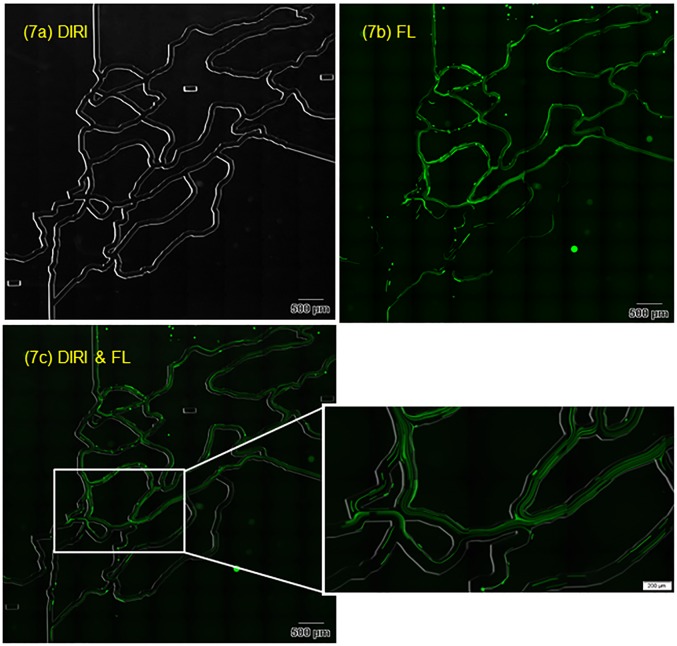
Photographs of fluorescent particles in the whole microfluidic channel. The exposure time for darkfield was 100 ms and that for confocal fluorescence was 810 ms. In total, 108 images were captured using a 10× objective lens, and were used to construct the WSI (whole-slide image). (7a) DIRI (Darkfield Internal Reflection Illumination) image. (7b) Confocal fluorescence image. (7c) Superimposed image of the fluorescence and DIRI images.

To assess the applicability of the system to fluorescent illumination, we observed fluorescent beads using DIRI and fluorescent illumination. We first observed the channel wall using DIRI with WSI mode (Fig. 7A) using a 10×, NA 0.4 objective lens. The software was programmed to construct a whole-slide image by acquiring and stitching together 108 individually acquired images. Next, we observed the trajectories of fluorescent beads by confocal microscopy in WSI mode. Fig. 7B shows the trajectories of fluorescent beads over 0.81 s with a flow rate of 1.2 μL/min. The image was obtained with fluorescent illumination (confocal) alone in this case. Note that the channel walls are difficult to identify. Fig. 7C shows superimposed DIRI and fluorescence images that were acquired sequentially. By superimposing the two images, tracer trajectories and channel geometry can be observed clearly. The long trajectories (visible as long green streaks) indicate beads moving at high velocity, whereas short trajectories (shorter green streaks) indicate low bead velocity. Locations that appear to have many lines indicate those with many moving beads. Thus, there are several advantages to this imaging technique: 1) the superimposed image provides information on the flow field in the whole microfluidic device, making measurements possible using channel architecture as fiduciary points; 2) this imaging modality does not require an expensive camera, such as those used frequently in high-speed imaging; and 3) high quality images can be obtained with a regular cooled CCD or sCMOS camera designed for fluorescence imaging.

**Fig 8:**
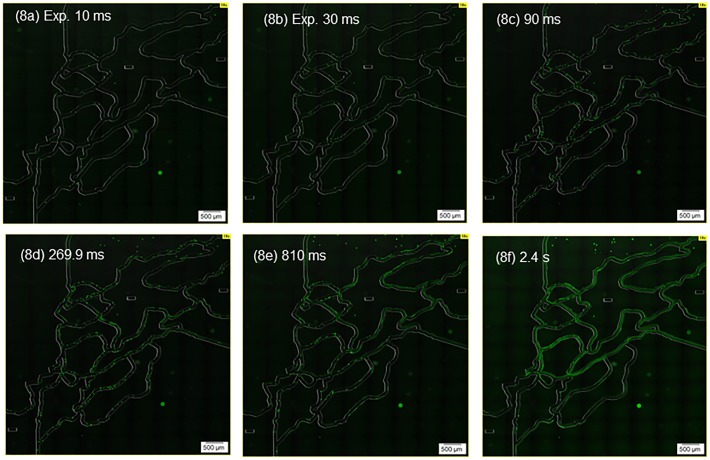
Photographs of exposure time changes for fluorescent particles in the whole microfluidic channel. Flow rate 1.2 μL/min. In total, 108 images were captured using a 10× objective lens, and were used to construct the WSI (whole-slide image). Images are superimposed the fluorescence and DIRI images. (8a) Exposure time 10 ms. (8b) Exposure time 30 ms. (8c) Exposure time 90 ms. (8d) Exposure time 269.9 ms. (8e) Exposure time 810 ms. (8f) Exposure time 2.4 s.

The velocity field in the microfluidic device can be roughly estimated by taking images with various exposure times, as shown in Fig. 8. The exposure times used in these images were 10 ms (Fig. 8A), 30 ms (8B), 90 ms (8C), 269.9 ms (8D), 810 ms (8E), and 2.4 s (8F). The velocity at each location can be roughly estimated by measuring the length of each bead trajectory and dividing it by the exposure time. This methodology is useful for determining whether the flow field of a microfluidic device is induced as designed.

**Fig 9:**
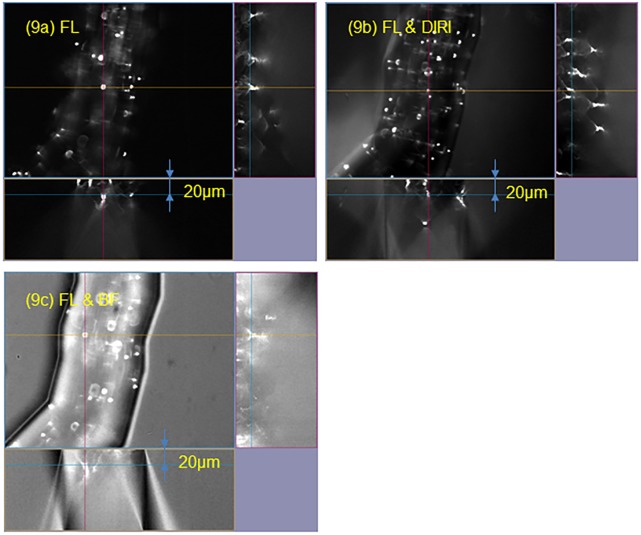
Three-dimensional images of fluorescent particles in microfluidic channels taken using confocal and DIRI. The image was taken with a 30× objective lens (NA 1.05, silicone oil immersion). (9a) Confocal fluorescence image. (9b) Image obtained by simultaneous using a confocal fluorescence excitation and DIRI (Darkfield Internal Reflection Illumination). (9c) Image obtained by simultaneous using confocal fluorescence excitation, and brightfield illumination.

The images shown in Fig. 9 were obtained by simultaneously using DIRI and fluorescent illumination. No flow was induced in this case, and the three-dimensional confocal image was obtained using a 30× silicone oil immersion objective lens (NA 1.05). In the case of fluorescent illumination alone (Fig. 9A), fluorescent beads could be observed clearly, but the edges of the channel were difficult to identify. When fluorescent and brightfield illumination were used simultaneously (Fig. 9C), the shape of the channel became clear, but the contrast of the fluorescent beads was lost because the background image was brighter. The bright background considerably reduced the contrast of the image. When DIRI and fluorescent illumination were used simultaneously (Fig. 9B), the shape of the channel could be observed clearly, and the contrast of the fluorescent beads was maintained. This technique can also be applied to dynamic phenomena, such as fluorescent beads flowing through the microchannel. A movie taken using simultaneous fluorescent and DIRI is available as supplementary data (S1 Video).

### 3. Combination of Multiple Fluorescence Imaging and DIRI Imaging

**Fig 10:**
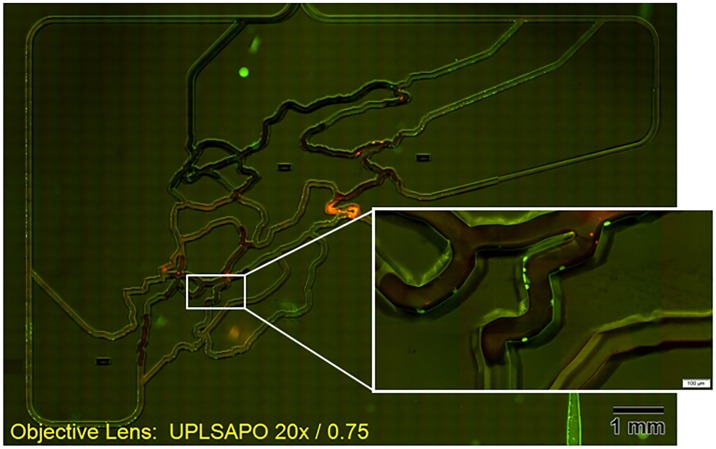
Endothelial and fibrosarcoma cells in the whole microfluidic channel. Endothelial cells (emitting green fluorescence) and fibrosarcoma cells (emitting red fluorescence) in the whole microfluidic device. The image was generated by superimposing two-color fluorescence images. The 20× objective lens (NA 0.75) was used.

Finally, we investigated the applicability of the present system for imaging cells within microfluidic devices. Endothelial cells (0.5 mL, 6×105 cells/mL) were injected into the microfluidic device, followed by incubation for 5 h at 37°C in a 5% CO2 atmosphere to allow the cells to attach to the walls of the microfluidic channel. Immediately before the experiment, fibrosarcoma cells (HT1080; 0.5 mL, 6×105 cells/mL) were injected into the microfluidic device. The endothelial cells were labeled with rhodamine 123, which was visualized in the green fluorescence emission channel, whereas the fibrosarcoma cells were labeled with MitoTracker Red CMXRos for visualization in the red fluorescence emission channel. A superimposed whole-slide image of green fluorescence, and red fluorescence images is shown in Fig. 10 (20× objective lens, NA 0.75). The system is capable of providing a high-contrast whole-slide image of two fluorescent colors.

**Fig 11:**
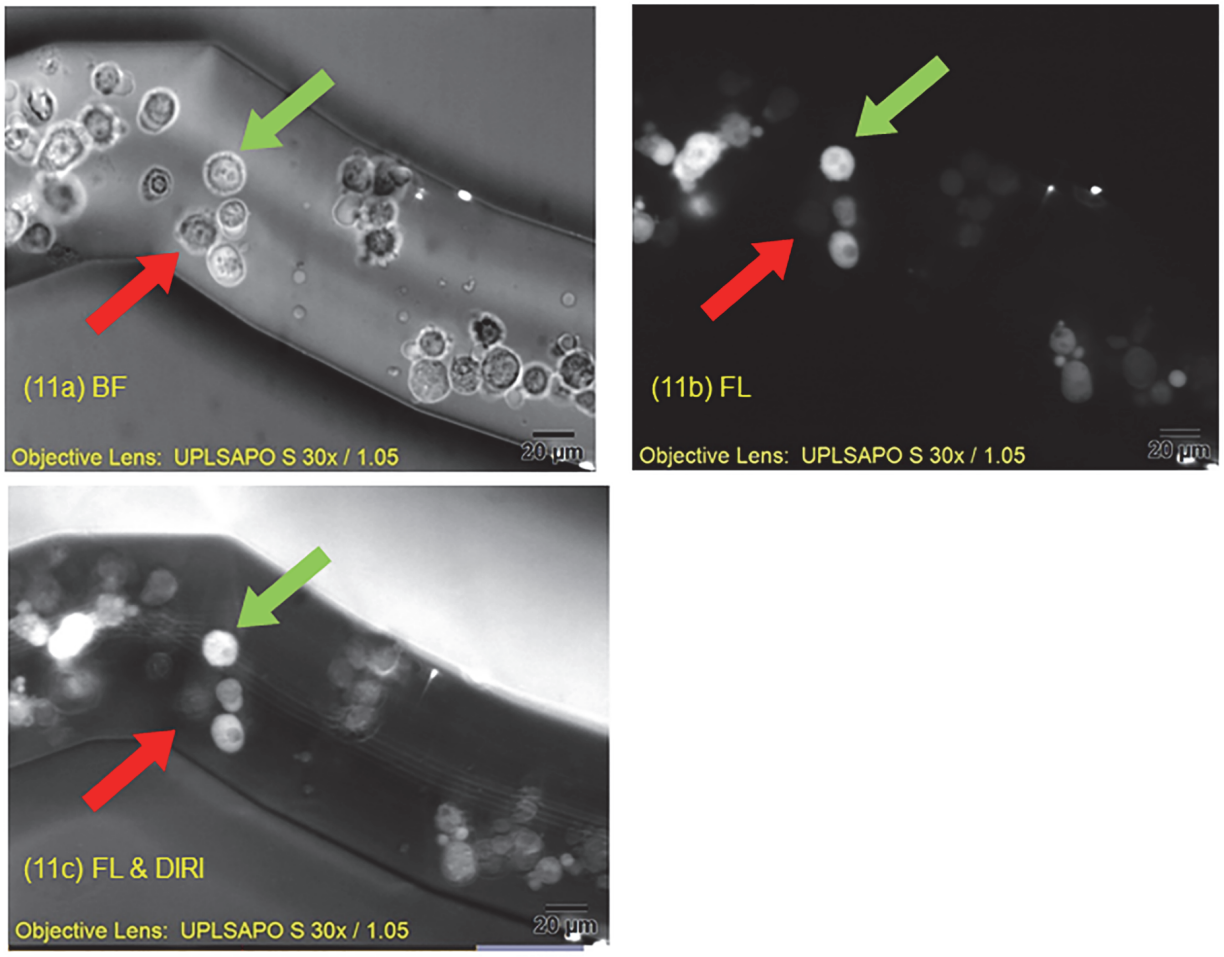
Images of cells obtained using three kinds of illumination. The images were captured using a 30× silicone oil immersion objective lens (NA 1.05). (11a) Brightfield image with an exposure time of 250 ms. (11b) Confocal fluorescence image with an exposure time of 200 ms. (11c) Image obtained by simultaneous using a confocal fluorescence excitation and DIRI (Darkfield Internal Reflection Illumination). The exposure time for fluorescence was 250 ms. The green arrow indicates an endothelial cell, and the red arrow a fibrosarcoma cell.

Fig. 11 shows the advantages of multiple channel fluorescence imaging plus DIRI imaging in certain types of experiment. These images show a magnified image of two types of stained cell acquired with a 30× silicone oil immersion objective lens (NA 1.05). In the brightfield image (Fig. 11A), high contrast can be seen at the edges of the channel walls, and the cells stand out well from the background. However, the difference between endothelial cells (green arrow) and fibrosarcoma cells (red arrow) is not obvious. Use of appropriate fluorescent illumination (Fig. 11B) enabled observation of one or the other population of cells, but rendered the walls of the microchannel difficult to see. In this image, the endothelial cells are fluorescing under blue excitation (green arrow) and can be seen clearly, but the edges of walls and the fibrosarcoma cells were unclear or not visible. Simultaneous observation of the green fluorescence emission and DIRI (Fig. 11C) facilitated observation of the endothelial cells (green arrow) and the edge of the channel walls. The high-contrast images were obtained using two types of illumination simultaneously, not by superimposing two independent images in post-processing. Thus, the present system is ideal for observing cell adhesion and invasion, in which dynamic interactions between multiple cell types and blood vessel walls can be visualized in a simplified in vitro model of a complicated in vivo situation.

## Conclusions

In this study, we investigated the applicability of using DIRI for observing a microfluidic device containing microbubbles, fluorescent particles, or fluorescently labeled cells. Whole-slide imaging was combined with these modalities to extend the capabilities of the system to allow multi-modal imaging of an entire microchannel device. Multi-modal imaging allowed clear visualization of both microbubbles and channel walls by utilizing DIRI and brightfield illumination simultaneously. The imaging methodology is useful not only for examination of static phenomena, such as clogging, but also for observation of dynamic phenomena, such as the detection of bubbles, fluorescent tracer particles, or labeled cells flowing through a channel. Observation of these dynamics makes possible a whole range of physical and physiological measurements of systems used for modeling more complex in vivo situations. This new multi-modal observation technique will make possible many previously difficult analyses, including observation of phenomena such as vessel wall adhesion of circulating cells under the moderation of pharmacological or physical agents, clot formation and inhibition, blood vessel obstruction, certain chemotaxis studies, the influence of turbulence or vessel walls, the influence of pharmacological moderators on circulating cell behavior, etc. The system developed here and the model validated in this study will be useful for a wide variety of engineering and biomedical applications using microfluidic devices.

## Supporting Information

S1 Video S1 Video is a movie of bubbles in the microfluidics system using brightfield and DIRI. This image was acquired using a 10× objective lens and an exposure time of 60 ms. Video obtained by simultaneous using brightfield illumination and DIRI (Darkfield Internal Reflection Illumination).
(AVI)
